# Infectious Diseases

**Published:** 1901-10

**Authors:** Alice M. Steeves

**Affiliations:** Chicago, Ill.


### INFECTIOUS DISEASES.[1]

BY ALICE M. STEEVES, D.D.S., CHICAGO, ILL.

A KNOWLEDGE of the possibility of transferring infectious diseases from one patient to another by means of instruments or otherwise, and of the character of the infection itself, is of the greatest practical value.

Of all diseases in which the infection is frequently carried from the mouth of one patient to that of another, perhaps the most important is syphilis. In this affection two different lesions are formed in or about the mouth, each characteristic of the disease and appearing at different periods in its course. The primary sore is frequently to be found upon the lips or within the mouth. This is especially true in children. The mucous patches of the secondary stage are usually seen in the mouth, where their seat is on the inner surface of the lip, the tongue, the palate, or the fauces. In size they are from an eighth to half an inch in diameter, and while they extend superficially they are never deep. They are whitish in color, with rounded edges, and are raised rather than depressed. These two lesions are the sources of most of the infection which causes new cases of the disease, the virus being carried by actual contact, surgical instruments, household utensils, or other accidental means, and to unaccustomed observers they may be overlooked or neglected or considered harmless because their true character is not recognized.

The frequency with which these conditions occur and the great danger attached to them should warn every oral surgeon to be ready to recognize them and to take proper precautions to protect himself and others.

The discovery that the germs of scarlet fever and diphtheria may be found in perfectly healthy mouths and throats, and the fact that these germs remain in the mouth and throat for long periods after the clinical signs of the disease have disappeared, suggest the probability that these diseases may be transferred from patient to patient by unclean instruments. Well-proved instances of this kind are indeed rare, yet this mode of infection is worthy of attention.

---

[1] Abstract of a paper read before the Section on Stomatology, American Medical Association, June, 1901.

The germs of diphtheria are sometimes found in tooth-cavities or in a healthy throat, and although they may not cause the disease in that individual because of his natural resistance, when carried to the mucous membrane of a susceptible person they may produce the disease in a virulent form. The bacillus of diphtheria may be the cause of all grades of sore throat, from a simple acute catarrh to an intense membranous inflammation extending into the nose and mouth, so that the true nature of a sore throat may not be recognized and the bacilli may be spread from patient to patient on septic instruments, as is so often done by means of infected spoons and forks.

Diphtheria bacilli usually remain in the throat two weeks after the membrane has disappeared, and have been found five or six weeks afterwards in some cases. The infectious nature of purulent discharges following scarlet fever, such as a rhinitis or pharyngitis, is now fully recognized, so that if after one of these infectious diseases catarrhal inflammations are present, the necessary precautions can be taken to prevent new cases of the disease.

In the same manner erysipelas, which begins so often at the juncture of the skin and mucous membrane, and pus cocci from suppurating processes in the mouth, may be transferred from patient to patient, and it is easily conceivable that the bacillus of tuberculosis could be taken from the mouth of a tubercular patient on dental instruments and be deposited in the mouth of another patient and then find its way into the lungs.

